# MiR-873-5p acts as an epigenetic regulator in early stages of liver fibrosis and cirrhosis

**DOI:** 10.1038/s41419-018-1014-y

**Published:** 2018-09-20

**Authors:** David Fernández-Ramos, Pablo Fernández-Tussy, Fernando Lopitz-Otsoa, Virginia Gutiérrez-de-Juan, Nicolás Navasa, Lucía Barbier-Torres, Imanol Zubiete-Franco, Jorge Simón, Agustín F. Fernández, Ander Arbelaiz, Ana M. Aransay, José Luis Lavín, Naiara Beraza, María J. Perugorria, Jesus M. Banales, Erica Villa, Mario F. Fraga, Juan Anguita, Matias A. Avila, Carmen Berasain, Paula Iruzibieta, Javier Crespo, Shelly C. Lu, Marta Varela-Rey, José M. Mato, Teresa C. Delgado, María L. Martínez-Chantar

**Affiliations:** 1 CIC bioGUNE, Centro de Investigación Cooperativa en Biociencias, Derio, Bizkaia Spain; 2 Centro de Investigación Biomédica en Red de Enfermedades Hepáticas y Digestivas (CIBERehd), Instituto de Salud Carlos III, Madrid, Spain; 3 Cancer Epigenetics Laboratory, Institute of Oncology of Asturias (IUOPA), HUCA, University of Oviedo, Oviedo, Spain; 4 Instituto de Investigación Sanitaria del Principado de Asturias (IISPA), Oviedo, Spain; 5 Department of Liver and Gastrointestinal Diseases, Biodonostia Health Research Institute—Donostia University Hospital—University of the Basque Country (UPV/EHU), San Sebastian, Spain; 6 Ikerbasque, Basque Foundation for Science, Bilbao, Spain; 7 Department of Gastroenterology, Azienda Ospedaliero-Universitaria & University of Modena and Reggio Emilia, Modena, Italy; 8 Nanomaterials and Nanotechnology Research Center (CINN-CSIC)—Universidad de Oviedo-Principado de Asturias, Oviedo, Spain; 9 Hepatology Programme, CIMA-University of Navarra, IdiSNA, Pamplona, Spain; 10 Department of Gastroenterology and Hepatology, Marqués de Valdecilla University Hospital. Infection, Immunity and Digestive Pathology Group, Research Institute Marqués de Valdecilla (IDIVAL), Santander, Spain; 11 Division of Digestive and Liver Diseases, Cedars-Sinai Medical Center, Los Angeles, CA USA

## Abstract

Glycine N-methyltransferase (GNMT) is the most abundant methyltransferase in the liver and a master regulator of the transmethylation flux. GNMT downregulation leads to loss of liver function progressing to fibrosis, cirrhosis, and hepatocellular carcinoma. Moreover, GNMT deficiency aggravates cholestasis-induced fibrogenesis. To date, little is known about the mechanisms underlying downregulation of GNMT levels in hepatic fibrosis and cirrhosis. On this basis, microRNAs are epigenetic regulatory elements that play important roles in liver pathology. In this work, we aim to study the regulation of *GNMT* by microRNAs during liver fibrosis and cirrhosis. Luciferase assay on the 3ʹUTR-*Gnmt* was used to confirm in silico analysis showing that *GNMT* is potentially targeted by the microRNA miR-873-5p. Correlation between *GNMT* and miR-873-5p in human cholestasis and cirrhosis together with miR-873-5p inhibition in vivo in different mouse models of liver cholestasis and fibrosis [bile duct ligation and *Mdr2 (Abcb4)*^*-/-*^ mouse] were then assessed. The analysis of liver tissue from cirrhotic and cholestatic patients, as well as from the animal models, showed that miR-873-5p inversely correlated with the expression of *GNMT*. Importantly, high circulating miR-873-5p was also detected in cholestastic and cirrhotic patients. Preclinical studies with anti-miR-873-5p treatment in bile duct ligation and *Mdr2*^-/-^ mice recovered GNMT levels in association with ameliorated inflammation and fibrosis mainly by counteracting hepatocyte apoptosis and cholangiocyte proliferation. In conclusion, miR-873-5p emerges as a novel marker for liver fibrosis, cholestasis, and cirrhosis and therapeutic approaches based on anti-miR-873-5p may be effective treatments for liver fibrosis and cholestatic liver disease.

## Introduction

Glycine N-methyltransferase (GNMT) is the most important and abundant S-adenosylmethionine (SAMe)-dependent methyltransferase in the liver. GNMT is predominantly expressed in hepatocytes although it is also found in other cell types such as cholangiocytes, the epithelial cells of the bile duct^1–3^. Lack of GNMT induces undesired methylation reactions leading to proliferative, inflammatory, and profibrogenic responses that culminate in liver disease^4,5^. GNMT expression is reduced in different liver diseases including liver cirrhosis of diverse etiology^1,6^, chronic cholestatic liver disease^7^, hepatocellular carcinoma (HCC)^1^, and cholangiocarcinoma^2^. In agreement, *Gnmt*-deficient (*Gnmt*^*-/-*^) mice, characterized by elevated SAMe levels, develop liver fibrosis spontaneously at the age of 3 months and HCC at 8 months^8^. Indeed, an aberrant DNA methylation signature has been identified in the *Gnmt*^*-/-*^ mouse^8^, underscoring the importance of this protein as an epigenetic regulator. Although *GNMT* promoter hypermethylation causes *GNMT* downregulation in some HCC patients^9^, other mechanisms are likely to be involved in the regulation of this gene in liver diseases such as fibrosis or cirrhosis.

Hepatic fibrosis is the result of the wound-healing response of the liver to repeated injury that occurs in most types of chronic liver diseases. The injury can be caused by the accumulation of lipids in the liver, as occurs during non-alcoholic fatty liver disease (NAFLD), can be a result of a toxicant or viral insult, such as excessive alcohol consumption or hepatitis, respectively, or the accumulation of bile acids (BA), as in chronic liver cholestasis. The finding of potential molecular and pathway targets for reverting or halting the progression of liver fibrosis or cholestasis to cirrhosis and HCC is an emerging field.

MicroRNAs (miRNAs) are highly conserved, small non-coding RNAs that post-transcriptionally regulate gene expression^10^ of essential biological processes, as well as cellular responses^11,12^. In the liver, miRNA signature has been implicated in NAFLD, cirrhosis, and liver cancer^13^. The biological significance and therapeutic potential of miRNAs in liver disease management is a rapidly growing field.

In this study, we show that liver fibrosis progression is associated with the repression of GNMT, which is targeted by miR-873-5p in two preclinical models of liver fibrosis and cholestasis: the bile duct ligation (BDL) and the *Mdr2* (*Abcb4*)-deficient mice (*Mdr2*^-/-^), respectively. Targeting miR-873-5p in these models resulted in a reduction of liver damage affecting mainly hepatocyte apoptosis and cholangiocyte proliferation, through a GNMT-dependent epigenomic mechanism. Of note, an inverse correlation between hepatic GNMT and miR-873-5p was identified in a cohort of cirrhotic patients with diverse etiology. Importantly, increased circulating miR-873-5p was also detected in serum samples from cirrhotic and cholestatic patients. A negative correlation was found between hepatic GNMT expression and serum levels of miR-873-5p in a cohort of cholestatic patients according with the fibrotic stage.

Overall, miR-873-5p emerges as a novel druggable target and a marker for liver fibrosis, cholestasis and cirrhosis.

## Results

### MiR-873-5p targets Gnmt expression in mouse hepatocytes

**Fig. 1:**
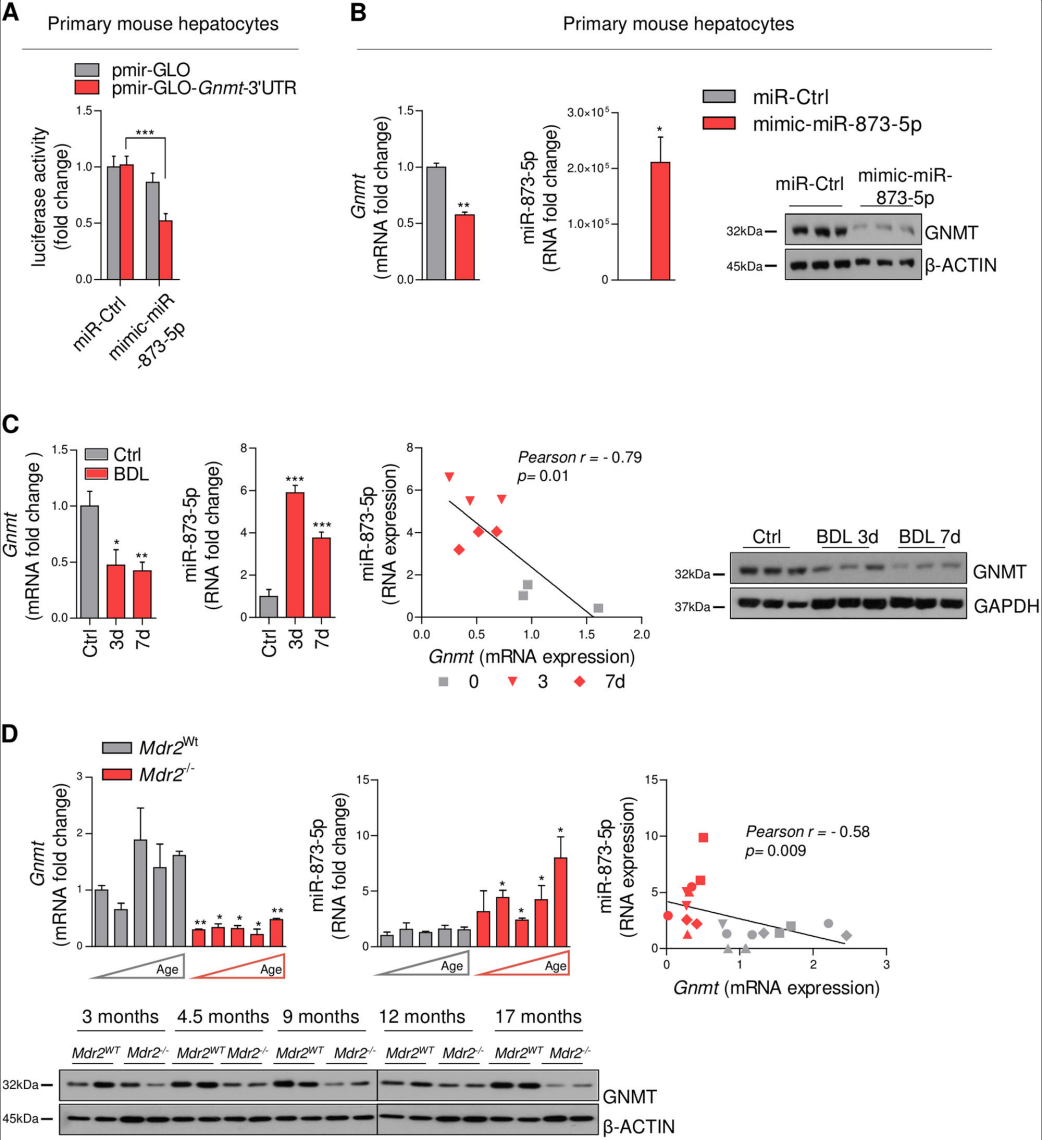
MiR-873-5p regulates GNMT expression by post-transcriptional repression and miR-873-GNMT levels are negatively correlated in mouse liver cholestasis. (**a**) Luciferase reporter assay of *Gnmt* 3ʹUTR expression in hepatocytes transfected with mimic-miR-873-5p (*N* = 8). (**b**) GNMT and miR-873-5p expression at mRNA and protein levels in primary hepatocyte after mimic-miR-873-5p transfection. *Gnmt*-miR-873-5p expression and correlation and WB analysis of GNMT at different time points of BDL (*N* = 3) (**c**) and in *Mdr2*^*WT*^ and *Mdr2*^*-/-*^ mice at different ages (3,4,9,12,17 months each column and 3▲, 4▼, 9♦, 12●, and 17■ months in the correlation panel) (*N* = 3) (**d**). Densitometry analyses of WB are shown in Supp. Fig. 1B, D, E. Data normalized as fold change vs. control. Error bars represent the means ± SEM. Statistical significance was determined by the Student’s *t*-test or ANOVA when more than two groups were compared. *p* < 0.05*; *p* < 0.01**; *p* < 0.001***

Understanding the mechanism underlying *GNMT* repression is essential for the development of new therapeutic approaches in liver fibrosis and cholestatic diseases. From three independent unbiased approaches employed (www.targetscan.org, www.ebi.ac.uk, and www.microrna.org) only miR-873-5p appears as a common microRNA targeting *GNMT* (Suppl. Fig. 1a). For functional analysis of miR-873-5p, primary mouse hepatocytes, characterized by high levels of GNMT, were transfected with pmir-GLO and pmir-GLO-*Gnmt*-3ʹUTR both fused to a luciferase reporter gene. Transfection with a miR-873-5p mimic (mimic-miR-873-5p) results in a 50% reduction of 3ʹUTR *Gnmt* reporter activity vs. those transfected with miR-Control (Fig. 1a). Moreover, mimic-miR-873-5p efficiently reduced mRNA and protein GNMT levels in cultured hepatocytes (Fig. 1b). Altogether, these data support miR-873-5p as a post-transcriptional repressor of *Gnmt*.

### MiR-873-5p inversely correlates with GNMT expression in liver fibrosis and cholestasis

The inverse association between *GNMT* and miR-873-5p expression was confirmed in different preclinical models of liver fibrosis. Administration of carbon tetrachloride (CCl_4_) for 6 weeks induced a reduction in *Gnmt* expression, associated with the induction of miR-873-5p (Suppl. Fig. 1C). Additionally, in two models of cholestatic liver disease induced by BDL after 3 and 7 days of surgery^14^ and the *Mdr2*^*-/-*^ mouse, a model of inflammation-induced cholestatic liver injury, fibrosis, and cancer^15^, we found an inverse correlation between GNMT levels and miR-873-5p expression (Fig. 1c, d).

**Fig. 2:**
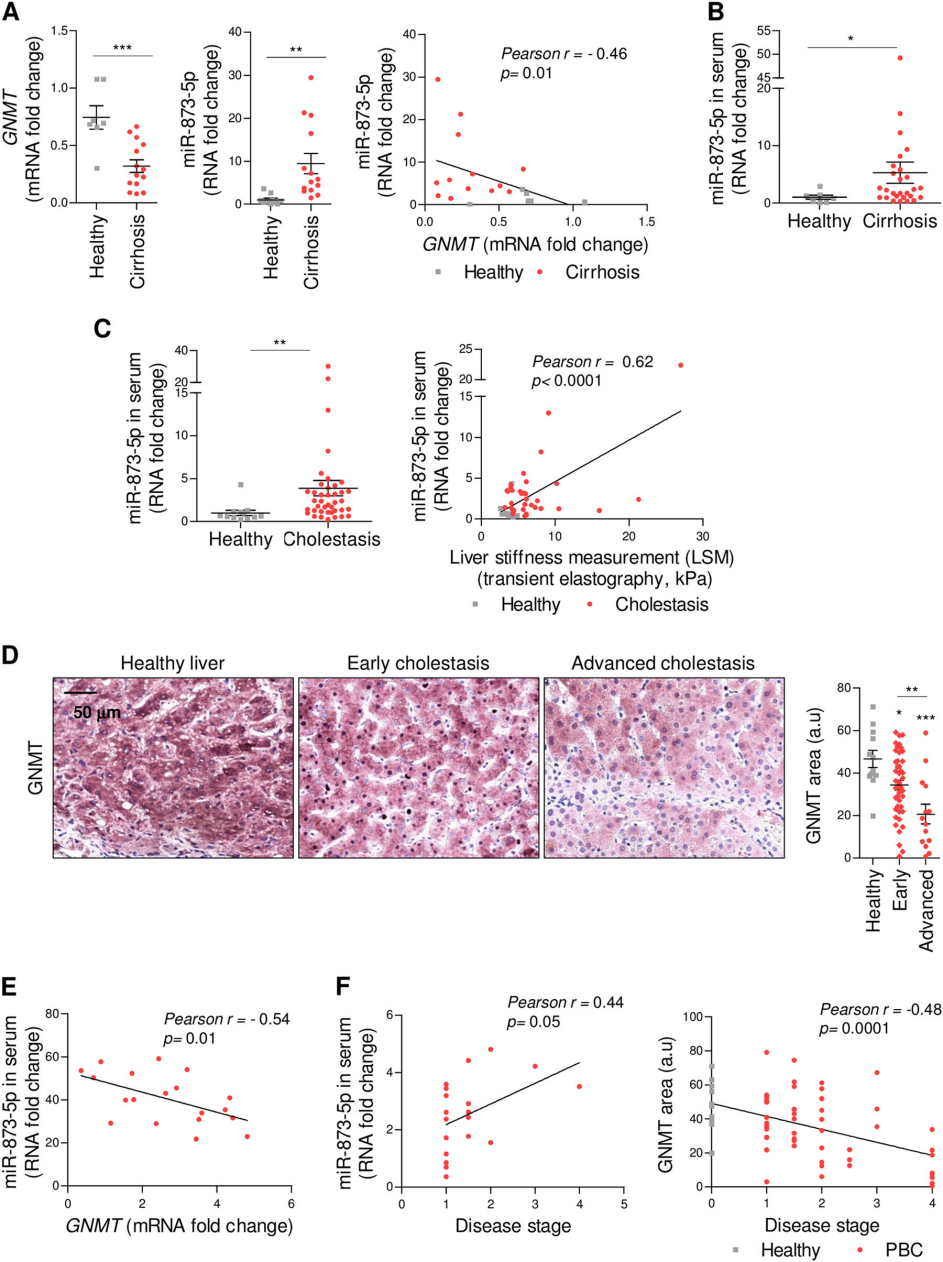
MiR-873-5p correlates with liver fibrosis development through the regulation of GNMT in the liver. Hepatic *GNMT*-miR-873-5p expression and correlation in a cohort of cirrhotic patients (*N* = 16) (**a**) and circulating miR-873-5p in cirrhotic patients (*N* = 35) (**b**). (**c)** Circulating miR-873-5p levels in cholestatic (PBC/PSC) patients (41) and its correlation with fibrosis (LSM, kPa). (**d**) GNMT levels in cholestatic patients (*N* = 64). (**e**) Correlation between circulating miR-873-5p and hepatic GNMT in cholestatic patients (*N* = 19). (**f**) Correlation between circulating miR-873-5p and hepatic GNMT with fibrotic stage in cholestatic patients, respectively. Data normalized as fold change vs. control. Error bars represent the means ± SEM. Statistical significance was determined by the Student’s *t*-test or ANOVA when more than two groups were compared. *p* < 0.05*; *p* < 0.01**; *p* < 0.001***

Likewise, an inverse correlation between hepatic GNMT expression and miR-873-5p was identified in a group of 16 cirrhotic patients of diverse etiology (Suppl. Table 1, Fig. 2a). These results prompted us to assay circulating miR-873-5p levels in cirrhotic patients. Blood samples were collected at the screening visit in 35 cirrhotic patients diagnosed with cirrhosis or advanced scaring (F4) with diverse etiology and levels of circulating miR-873-5p were evaluated and compared to nine healthy controls. Higher miR-873-5p levels in serum were observed in cirrhotic patients (Fig. 2b). Subsequently, we analyzed circulating miR-873-5p levels in a cohort of cholestatic patients primary biliary cholangitis (PBC)/ primary sclerosing cholangitis (PSC) (*n* = 41) and compared to healthy patients with antimitochondrial antibodies (AMA + ) but without abnormal liver test (Suppl.Table II). In these patients, whose circulating miR-873-5p levels are increased relative to healthy controls, a positive correlation was also found with fibrosis grade (Fig. 2c). Furthermore, a reduction of hepatic GNMT expression was associated with the fibrosis grade in a cohort of 64 liver cholestasis patients (Suppl. Table III and Fig. 2d). Finally, a significant correlation between reduced expression of hepatic GNMT and increased levels of miR-873-5p in the serum of a cohort of patients with early and advance cholestatic liver disease was detected in 19 paired samples (Fig. 2e). Fibrotic stage positively correlated with miR-873-5p and negatively correlated with GNMT levels (Fig. 2f). Overall, miR-873-5p expression is augmented and inversely correlated with hepatic GNMT expression in cirrhosis and cholestatic disease.

### Anti-miR-873-5p attenuates liver injury after bile duct ligation

**Fig. 3:**
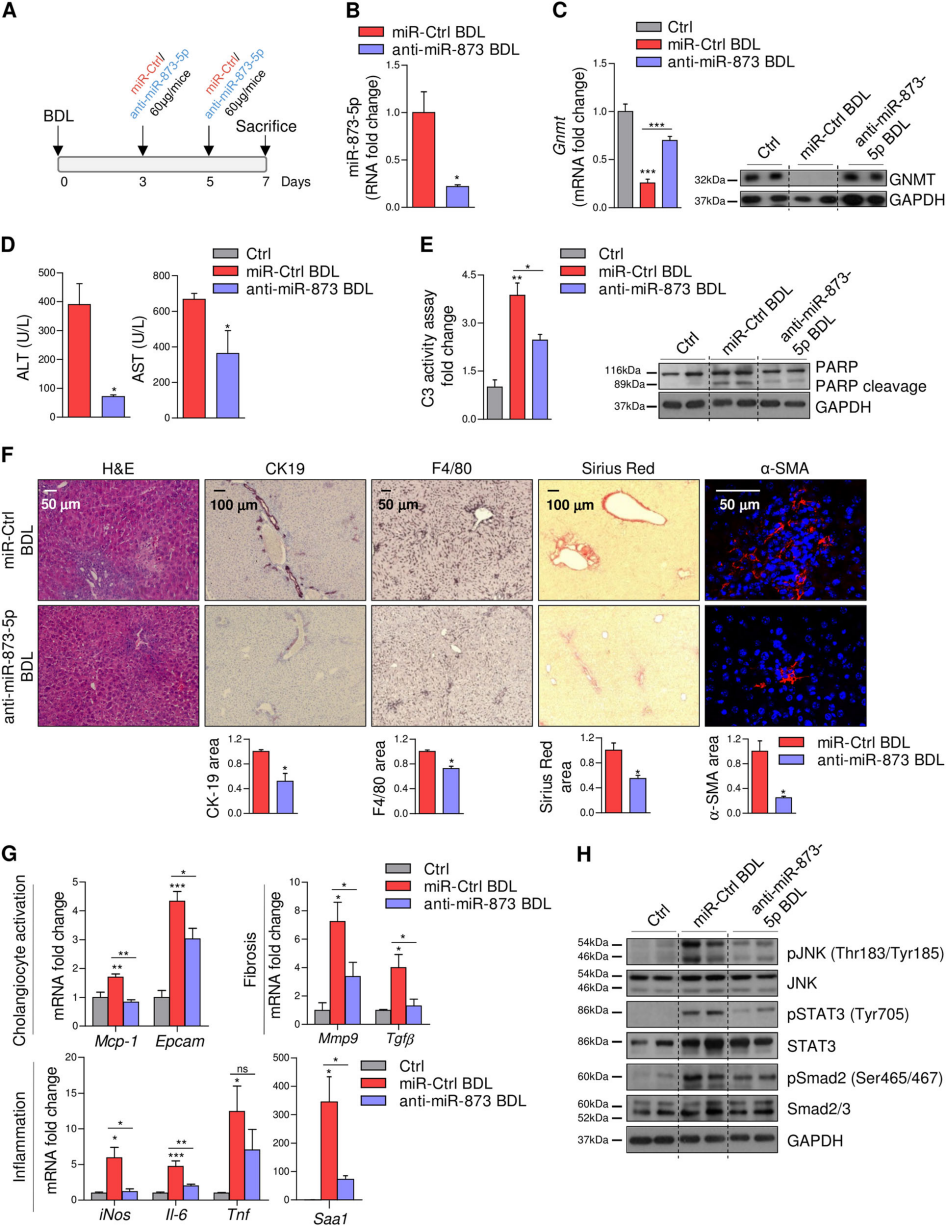
Anti-miR-873-5p attenuates BDL-induced liver injury through GNMT regulation. (**a**) Time scheme of the BDL mouse model with anti-miR-873-5p (time points indicated). MiR-873-5p expression (**b**) and GNMT mRNA and protein expression (**c**) in the liver of miR-Ctrl-BDL and anti-miR-873-5p-BDL mice. (**d**) Serum transaminases (ALT and AST) levels in miR-Ctrl and anti-miR-873-5p-treated-mice at 7 days of BDL. (**e**) Caspase 3 activity and WB analysis of apoptosis mediated by PARP cleavage. (**f**) H&E, F4/80, Sirius Red, αSMA, and CK19 staining in liver sections from miR-Ctrl and miR-873-5p-inhibited-mice at 7 days of BDL. (**g**) qPCR analysis of indicated genes in total liver extracts at 7 days of BDL. (**h**) WB analysis with indicated Ab in total liver of WT, BDL-Ctrl and anti-miR-873-BDL mice at 7 days. Densitometry analyses of WB are shown in Supp. Fig. 2B. Data normalized as fold change vs. control. Error bars represent the means ± SEM (*N* = 4). Statistical significance was determined by the Student’s *t*-test or ANOVA when more than two groups were compared. *p* < 0.05*; *p* < 0.01**; *p* < 0.001***

The potential benefit of targeting miR-873-5p was evaluated in the BDL animal model of cholestasis. Thus, we performed BDL surgery for one week in *Gnmt* wild-type (WT) mice and injected anti-miR-873-5p or a miR-Ctrl i.v. at day 3 and 5, corresponding to the initial phase of hepatic fibrogenesis^14^ (Fig. 3a and Suppl Fig. 2A) and sacrificed animals at 7 days after BDL. MiR-873-5p inhibition (Fig. 3b) resulted in GNMT restoration levels (Fig. 3c). No effect on miR-873-5p and *Gnmt* mRNA levels was detected in other tissues where *Gnmt* is usually expressed, such as pancreas and kidney, after miR-873-5p inhibition (data not shown). Anti-miR-873-5p treatment was associated with reduced serum transaminases (ALT/AST), caspase 3 activity and PARP cleavage as well as parenchymal disruption as detected by H&E staining, all readouts of cell death (Fig. 3d–f).

In the BDL mouse model, cholangiocyte proliferation and liver inflammation play essential roles during the progression of fibrosis that is linked to BA-induced hepatocyte cell death. Anti-miR-873-5p treatment in vivo significantly reduced the proliferation of cholangiocytes as evaluated by cytokeratin 19 (CK19) levels (Fig. 3f) and counteracted the expression of markers related with cholangiocyte activation, such as monocyte chemoattractant protein (*Mcp-1*) and epithelial cell adhesion molecule (*Epcam*) (Fig. 3g). Moreover, miR-873-5p inhibition minimized inflammatory markers such as F4/80, inducible nitric oxide synthase (*iNos*), acute phase response gene serum amyloid A1 (*Saa1*), interleukin-6 (Il-6), and JNK phosphorylation (Fig. 3f–h). Indeed, tumor necrosis factor (*Tnf*) and *Il-6* reduction (Fig. 3g) modulated STAT3 activation after miR-873-5p inhibition (Fig. 3h). Finally, in miR-873-5p-inhibited BDL mice, fibrogenic indicators such as Sirius red, alpha smooth muscle actin (*α-SMA*), metalloproteinase 9 (*Mmp9*), transforming growth factor beta (*Tgfb*), and Smad2/3 phosphorylation were significantly reduced in comparison to those mice treated with miR-Control (Fig. 3f–h).

RNA sequencing analysis further retrieved a completely different gene ontology (GO) profile in the BDL model after miR-873-5p inhibition. During this initial phase of hepatic fibrogenesis, biological processes related to cell proliferation, natural killer cell activation, and immune response processes were downregulated in anti-miR-873-5p vs. miR-Ctrl mice. On the other hand, anti-miR-873-5p-treated-mice showed increased gene expression related to biological processes implicated in steroid and fatty acid metabolism and in the respiratory electron transport chain compared to miR-ctrl mice (Supp. Fig. 3A and Supp. Table IV and V).

Of relevance, the treatment of *Gnmt*^-/-^ mice after 3 and 5 days of BDL with anti-miR-873-5p failed to counteract both inflammation and fibrosis (Supp Fig. 4a), indicating that *Gnmt* is one of the principal targets that mediates the anti-miR-873-5p effect in vivo, even though miR-873-5p could affect many different mRNAs besides *Gnmt*.

Overall, these data show that miR-873-5p inhibition is hepatoprotective and reduces cell death, affects ductular reaction and prevents the generation of liver inflammation and fibrosis after BDL, which is suggested to take place by enhancing GNMT expression.

### MiR-873-5p directly regulates hepatocyte apoptosis and cholangiocyte proliferation

In the pathology of liver fibrosis and in cholestatic liver injury different types of hepatic cells including hepatocytes, cholangiocytes, inflammatory macrophages (Kupffer cells)^16^ and Natural Killer^7,17–19^ cells, and hepatic stellate cells (HSC)^20^ are known to mediate different effects, regulating the development and the progression of the disease. During initiation of fibrogenesis, hepatocyte apoptosis and cholangiocyte proliferation are the primary events driving disease progression^14^. Although GNMT in the liver is known to be mainly found in hepatocytes, it has been described to be expressed in other cell types such as cholangiocytes, where its expression is associated with a differentiated and a non-proliferative phenotype of these cells^2^. Thus, we have analyzed the targeting of *Gnmt* by miR-873-5p in these cells and its potential contribution to cholestatic liver injury.

**Fig. 4:**
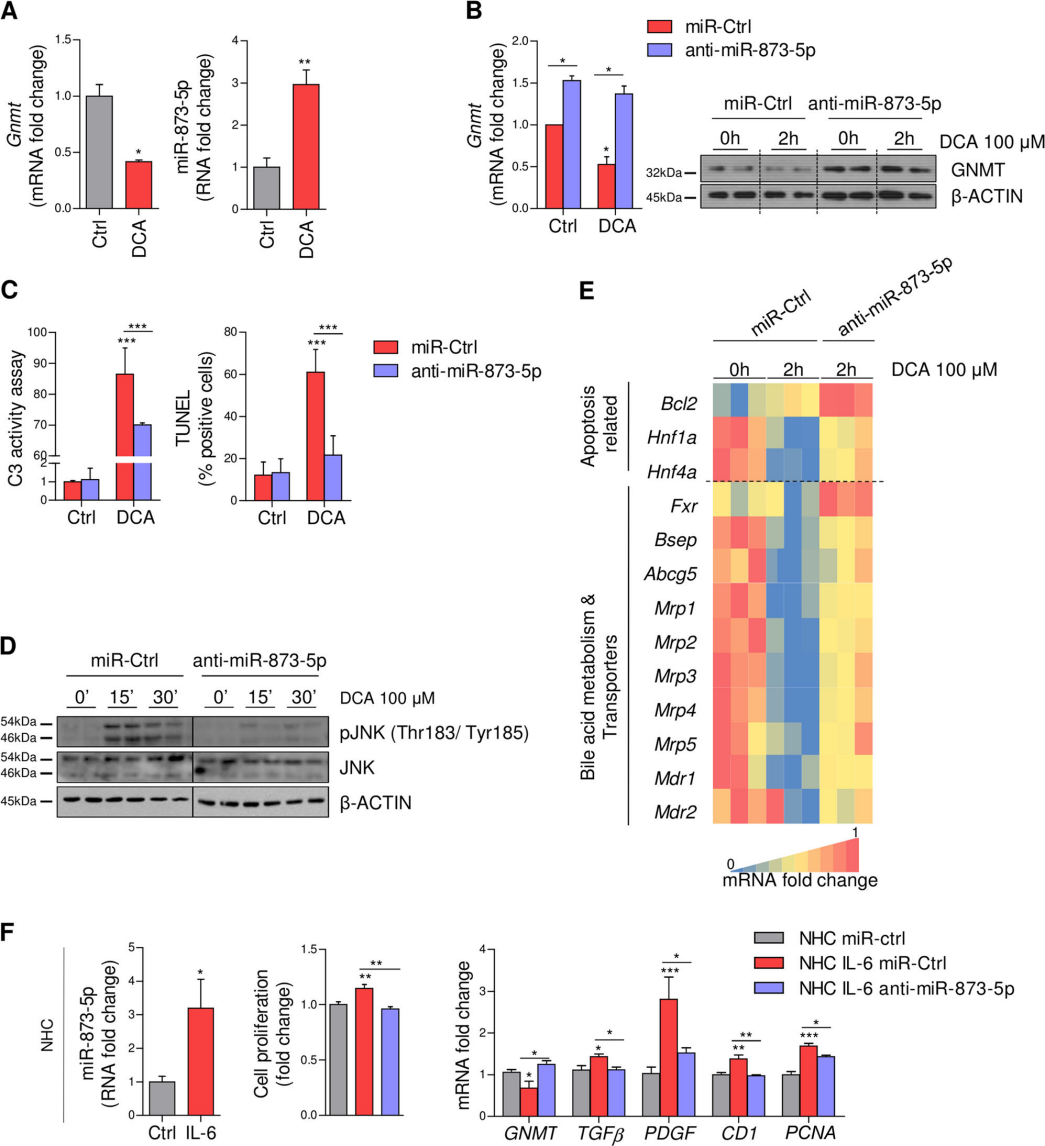
MiR-873-5p regulates hepatocyte apoptosis and cholangiocyte proliferation by targeting GNMT. (**a**) *Gnmt* and miR-873-5p expression in cultured hepatocytes treated with deoxycholic acid (DCA) for 2 h (*N* = 3). (**b**) GNMT expression analysis by qPCR and WB after anti-miR-873-5p and DCA treatment. Cell death was measured by C3 activity (2 h) TUNEL and JNK phosphorylation (15’/30’) at different time points of DCA treatment (**c**, **d**). (**e**) Gene expression analysis by qPCR of indicated genes implicated in BA-induced apoptosis, metabolism and transport after 2 h of DCA and anti-miR.873-5p treatment (*N* = 3). Densitometry analyses of WB are shown in Supp. Fig. 5A. (**f**) MiR-873-5p expression in normal human cholangiocytes (NHC) after 48 h of Interleukin-6 (IL-6) treatment (*N* = 6). Cell proliferation measured by MTT assay and analysis of *GNMT* and indicated genes measured by qPCR in anti-miR-873-5p cholangiocytes treated with IL-6 (*N* = 4). Data normalized as fold change vs. control. Error bars represent the means ± SEM. Statistical significance was determined by the Student’s *t*-test or ANOVA when more than two groups were compared. *p* < 0.05*; *p* < 0.01**; *p* < 0.001***

Firstly, primary isolated murine hepatocytes were cultured with the toxic bile acid deoxycholic acid (DCA, 100 µM) for 2 h to induce hepatocyte apoptosis, an in vitro cell model that mimics in vivo BDL. Under these circumstances, DCA treatment was associated with reduced *Gnmt* expression together with the induction of miR-873-5p and hepatocyte apoptosis, suggesting the importance of the regulation of GNMT-miR-873-5p axis in hepatocytes (Fig. 4a). The potential role of miR-873-5p in the induction of BA-induced apoptosis in hepatocytes through the repression of *Gnmt* was studied by inhibiting miR-873-5p directly in these cells. MiR-873-5p inhibition in hepatocytes induced GNMT levels (Fig. 4b), resulting in attenuation of BA-induced apoptosis measured by Caspase 3 activity, TUNEL assay and JNK phosphorylation (Fig. 4c, d). Moreover, miR-873-5p inhibition increased the expression of different genes related to BA-induced apoptosis [B-Cell CLL/Lymphoma 2 (*Bcl2*) and the Hepatocyte nuclear factor (*Hnf*) *Hnf1α* and *Hnf4α*] and, more importantly, genes implicated in BA metabolism and regulation, such as the master regulator of BA metabolism *Fxr* and different BA exporters, such as *Bsep, Abcg5* and some *Mdr/Mrp* (Fig. 4e).

Then, GNMT targeting by miR-873-5p was evaluated in cholangiocyte cell lines. It has been reported that inflammatory mediators like IL-6 (significantly augmented after BDL and modulated in the presence of anti-miR-873-5p (Fig. 3g)) induces cholangiocyte proliferation. Indeed, the normal human cholangiocyte cell line NHC incubated with IL-6 show increased miR-873-5p expression (Fig. 4f). MiR-873-5p inhibition reduced cholangiocyte proliferation measured by MTT assay and the levels of cyclin D1 (*CD1*) and proliferating cell nuclear antigen (*PCNA*) (Fig. 4f). Furthermore, anti-miR-873-5p increased *GNMT* expression and minimized the levels of growth factors (*TGFβ* and platelet derived growth factor (*PDGF*)) implicated in cholangiocyte contribution to BDL-induced fibrosis (Fig. 4f).

Overall, these data suggest that anti-miR-873-5p has broad effects in liver cells affecting fibrosis progression, mainly mediating anti-apoptotic activity in hepatocytes and anti-proliferative effect in cholangiocytes contributing to reduce liver injury and fibrosis progression.

### Anti-miR-873-5p prevents liver injury in Mdr2^-/-^ mice

**Fig. 5:**
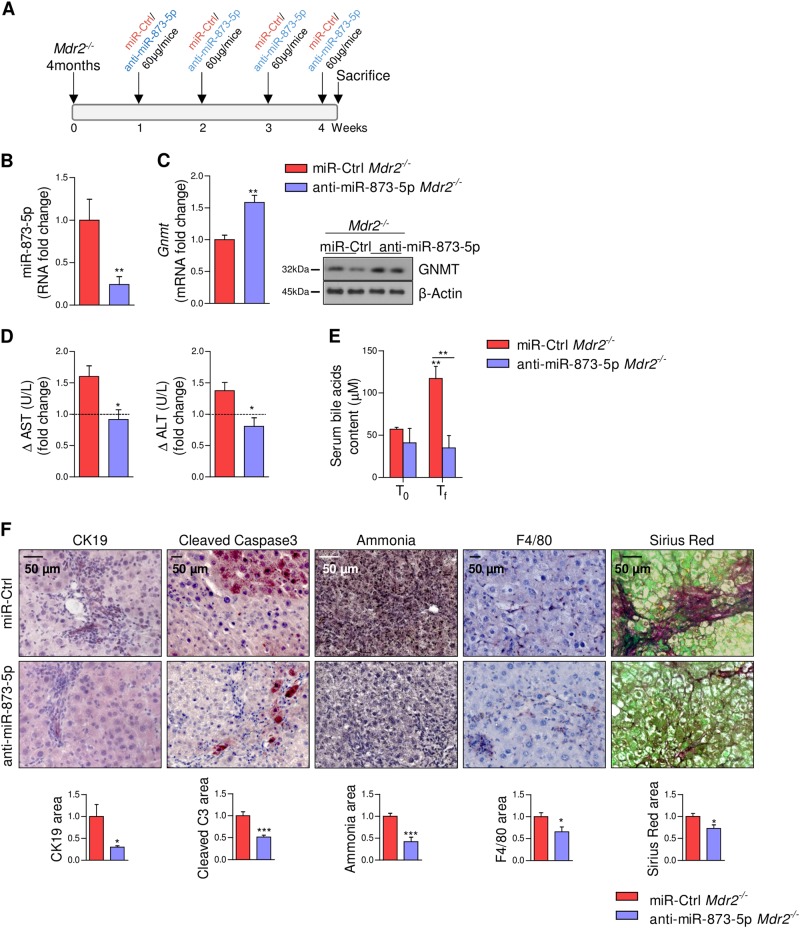
Anti-miR-873-5p decreases hepatocyte apoptosis, cholangiocyte proliferation, inflammation, and fibrogenesis in Mdr2^*-/-*^ mice. (**a**) Time scheme of the anti-miR-873-5p treatment in Mdr2^*-/-*^ mice (time points indicated). MiR-873-5p (**b**) and GNMT expression (**c**) in the liver of *Mdr2*^*-/-*^ and anti-miR-873-*Mdr2*^*-/-*^ mice. (**d**) Serum transaminases levels expressed as fold change from the initial values and (**e**) bile acid content in *Mdr2*^*-/-*^ mice. (**f**) CK19, Cleaved Caspase 3, ammonia, F4/80, and Sirius red staining in liver sections from miR-Ctrl and miR-873-5p-inhibited *Mdr2* ^*-/-*^ mice. Densitometry analyses of WB are shown in Supp. Fig. 6C. Data normalized as fold change vs. control. Error bars represent the means ± SEM (*N* = 5). Statistical significance was determined by the Student’s *t*-test or ANOVA when more than two groups were compared *p* < 0.05*; *p* < 0.01**; *p* < 0.001 ***. ----- line in **d** indicates initial levels of the measurements

Mice lacking the ATP-binding cassette ABCB4 protein encoded by the *Mdr2* gene (*Mdr2*^*-*/-^) provide a model for the study of cholestasis in the context of chronic inflammation as a result of increased BA accumulation^15^. The increase in miR-873-5p and the low expression levels of GNMT at the different stages of hepatic fibrogenesis in the *Mdr2*^-/-^ mice (Fig. 1d) prompted us to investigate the impact that anti-miR-873-5p treatment could have in this model. Four-month old *Mdr2*^-/-^ mice, with advanced fibrosis and biliary proliferation (Suppl Fig. 6a), were treated once a week with anti-miR-873-5p during 4 weeks (Fig. 5a). Anti-miR-873-5p treatment rescued GNMT (Fig. 5b, c) and reduced ALT/AST transaminases, total serum BA levels (Fig. 5d, e), cholangiocytes proliferation by CK19, hepatocyte death assessed by caspase 3 activity and hepatic ammonia content^21^ (Fig. 5f). Moreover, *Mdr2*^-/-^ mice treated with anti-miR-873-5p showed lower cell death receptor *Dr5* and higher biliary acids transporters of the ABC superfamily *Mdr1, Mrp3, and Mrp5* (Suppl Fig. 6B), consistent with the reduction of BA content, BA-induced apoptosis and markers related to cholangiocyte activation (Suppl Fig. 6B). These changes were accompanied by a reduced inflammatory response as determined by F4/80 and *Il-6*, *Ccl1* and *iNos* expression, as well as less fibrosis evaluated through Sirius red staining and the levels of profibrogenic (*Tgfb*, *Timp1*, and *Timp2*) genes (Fig. 5f and Suppl Fig. 6b). Altogether, these data suggest that anti-miR-873-5p targeting in the Mdr2-/-cholestasis animal model reduces cholangiocyte proliferation, increases the export of bile acids and ameliorates BA-induced hepatocyte cell death and inflammatory response.

### MiR-873-5p regulation results in GNMT-dependent epigenomic modulation

**Fig. 6:**
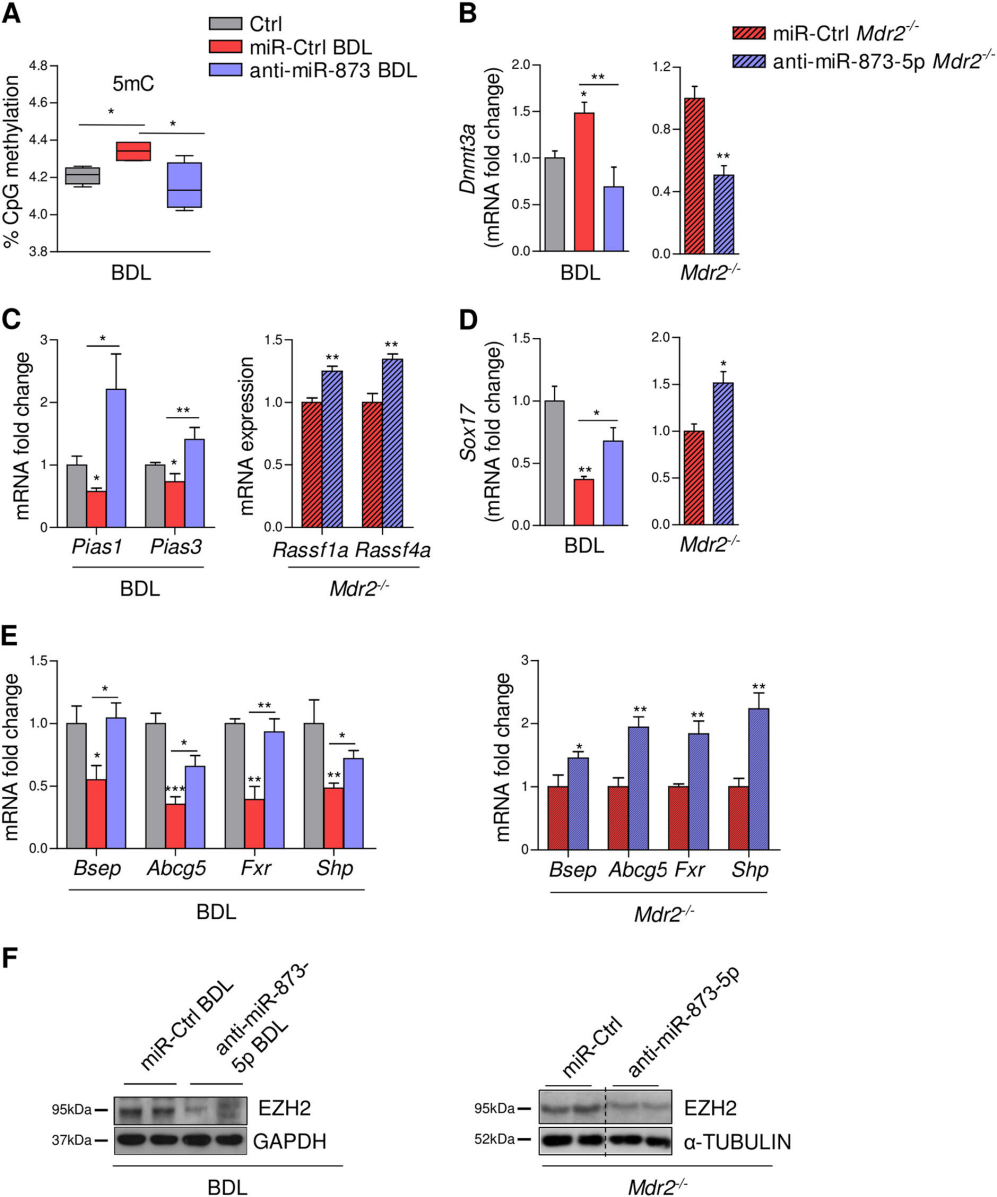
Epigenomic alterations contributing to liver fibrosis are reverted in anti-miR-873-5p-treated-mice through the restoration of GNMT, SAMe metabolism, and transmethylation flux. (**a**) DNA CpG (5mC) methylation in Ctrl and BDL/anti-miR-873-BDL livers (*N* = 4). qPCR and WB analysis of genes implicated in DNA methylation (**b**). qPCR analysis of indicated genes susceptible of promoter hypermethylation related to cholangiocyte proliferation and bile acid metabolism (**c**-**e**) in miR-Ctrl and anti-miR-873-5p BDL/*Mdr2*^*-/-*^ mice (*N* = 4 (BDL) and *N* = 5 (*Mdr2*^*-/-*^). (**f**) WB analysis of EZH2 in anti-miR-873-5p-BDL/*Mdr2*^*-/-*^ mice (*N* = 4 (BDL) and *N* = 5 (*Mdr2*^*-/-*^). Densitometry analyses of WB are shown in Supp. Fig. 7C. Data normalized as fold change vs. control. Error bars represent the means ± SEM. Statistical significance was determined by the Student’s *t*-test or ANOVA when more than two groups were compared. *p* < 0.05*; *p* < 0.01**; *p* < 0.001***

DNA (CpG) methylation acts as a blueprint for global alterations in the epigenome that drive liver injury^22^ among them hepatic fibrogenesis^23–26^. BDL and *Mdr2*^-/-^ mice under anti-miR-873-5p treatment showed improved hepatic SAMe metabolism with a decrease in the ratio SAMe/S-adenosylhomocysteine (SAH) consistent with the re-expression of GNMT (Table 1). This effect in the methylation flux correlated with a reduction in the global DNA methylation content as detected after miR-873-5p inhibition (Fig. 6a). DNA methylation is mediated by two groups of DNA methyltransferases (DNMTs), DNMT1 that is implicated in maintenance of DNA methylation and DNMT3a/3b that are responsible for de novo DNA methylation^27^. According to the changes in DNA methylation levels in the liver, anti-miR-873-5p treatment reduced *Dnmt3a* expression in both fibrotic animal models (Fig. 6b). Indeed, the inhibition of miR-873-5p during BDL and in *Mdr2*^-/-^ mice increased the expression of other genes of the methionine cycle besides *Gnmt*, such as Methionine adenosyltransferase (*Mat1a*) and S-adenosylhomocysteine hydrolase (*Sahh*) (Suppl. Fig. 7A).

**Table 1:** Liver SAMe and SAH levels regulation by anti-miR-873-5p in BDL and *Mdr2*^*-/*-^ mice

GROUP	SAMe (pmol/mg prot)	SAH (pmol/mg prot)	SAMe/SAH (pmol/mg prot)	*T*-TEST SIGNIFICANCE (SAMe/SAH ratio)
BDL MICE				
Wt	109.06 ± 42.36	63.22 ± 12.03	**1.75** ± 0.72	
miR-Ctrl BDL	158.61 ± 34.32	41.30 ± 13.93	**4.00** ± 1.11	**0.042** ^a^
Anti-miR-873-5p-BDL	108.80 ± 38.99	58.97 ± 7.57	**1.86** ± 0.67	**0.046** ^b^
*Mdr2*^*-/-*^ MICE				
miR-Ctrl	61.81 ± 4.35	2.55 ± 0.41	**21.12** ± 2.75	
Anti-miR-873-5p	53.03 ± 10.22	3.27 ± 06.8	**16.58** ± 3.19	**0.034** ^c^

^a^*p* CTRL vs. miR-Ctrl BDL

^b^*p* miR-Ctrl BDL vs. anti-miR-873-5p BDL

^c^*p* Mdr2^-/-^ miR-Ctrl vs. anti-miR-873-5p

Different genes could be regulated by methylation of their CpG islands. The activity of STAT3, a key player in liver inflammation, was repressed in BDL under miR-873-5p inhibition (Fig. 3g), correlating with the re-expression of its inhibitor Protein inhibitor of activated STAT (*Pias*) *Pias 1/3* (Fig. 6c), that has been associated to its promoter methylation status^28^. Similarly, the promoter regulation of RAS-association domain family (RASSF) (inhibitors of RAS signaling), has been reported to be susceptible of hypermethylation in the absence of GNMT^8^ and in early HCCs^28^. We have found that the expressions of RASSF genes (*Rassf1a*/*Rassf4a*) are upregulated in *Mdr2*^-/-^ mice after blockade of miR-873-5p (Fig. 6c).

Regulation of cholangiocyte proliferation and BA metabolism have both been described to be under epigenetic control^29,30^. SRY-Box 17 (SOX17) regulates the phenotype of normal human cholangiocytes acting as a tumor suppressor in cholangiocarcinoma, occurring its downregulation through DNA methylation^31^. Under anti-miR-873-5p treatment, *Sox17* expression was significantly upregulated in BDL and *Mdr2*^*-/-*^ mice (Fig. 6d). Finally, inhibiting miR-873-5p also restored the expression of genes methylated under pathological conditions, such as *Abcg5* and *Bsep*, as well as the nuclear receptors *Shp* and *Fxr*, implicated in BA transport and metabolism^29^ (Fig. 6e).

Besides DNA, SAMe can methylate histones at different residues. Histone modification is also involved in establishing patterns of gene repression during liver injury. Methylation of histones and DNA are linked to the activation of HSCs^32^. Importantly, miR-873-5p inhibition significantly reduced the protein levels of EZH2, a profibrogenic histone lysine methyltransferase, after BDL and in *Mdr2*^-/-^ mice (Fig. 6f). Also, the mRNA level of the histone methyltransferase *Ash1* is reduced after anti-miR-873-5p treatment, correlating with the decrease in the methylation level of its target H3K4, which has been related with TGFβ induction in fibrosis (Suppl. Fig. 6B)^33^.

**Fig. 7:**
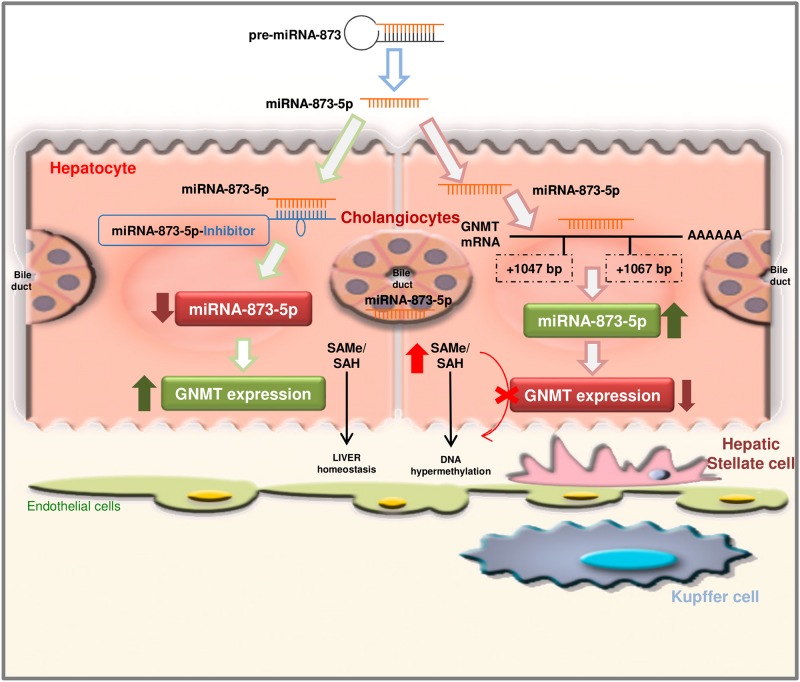
**MiR-873-5p targets GNMT expression in the liver mediating global epigenomic changes and contributing to liver fibrosis progression**

Overall, these data suggest that the prompt recuperation of hepatic *GNMT* expression through anti-miR-873-5p treatment could lead to the restoration of SAMe metabolism and the epigenetic regulation of specific genes that are implicated directly in BA homeostasis, cholangiocyte proliferation and inflammatory and fibrogenic pathways (Fig. 7).

## Discussion

GNMT is considered a major regulator of the transmethylation flux in the liver controlling liver homeostasis and health^4,5^. A decrease in GNMT levels occurs in NAFLD, at early stages of fibrosis, cirrhosis, and HCC^1,6,7,34,35^. Data highlighting that attenuation of GNMT was found at early stages of liver disease, where fibrosis is still not prominent, points to GNMT deficiency as a contributor/mediator of fibrogenesis rather than a consequence. Even though *GNMT* repression in some primary tumors has been associated with aberrant promoter methylation^9^, hypermethylation does not seem to be the unique mechanism for *GNMT* regulation, and it may coexist with other regulatory pathways^9^.

miRNAs regulate a wide variety of biological functions in the liver and are directly implicated in inflammation, cirrhosis, and malignant transformation^13^. These small molecules are, therefore, emerging as viable therapeutic tools. Three independent unbiased approaches revealed miR-873-5p as a potential microRNA targeting *GNMT*. In here, we have shown that miR-873-5p is modulated in several in vitro and in vivo models of liver fibrosis where GNMT levels are modified. Furthermore, in the clinical setting, we have found high levels of circulating miR-873-5p in the serum of cirrhotic patients as well as a negative correlation between hepatic *GNMT* and miR-873-5p in human cirrhosis and cholestasis. Altogether, this evidence allowed us to identify for the first time GNMT as a direct target of miR-873-5p.

Although liver fibrosis was historically thought to be an irreversible process, the understanding of its molecular pathogenesis has stimulated the development of novel antifibrotic therapies, still in an experimental phase^36^. Increased miR-873-5p levels have been observed in hepatocytes and in proliferative cholangiocytes under cholestatic/fibrogenic situations. Anti-miR-873-5p treatment in vitro decreased the BA-induced hepatocyte cell death as well as cholangiocyte proliferation. Importantly, anti-miR-873-5p treatment in *Gnmt* wild-type mouse submitted to BDL surgery blocked the overwhelming inflammatory profile associated to fibrosis. Of relevance, no effects under miR-873-5p inhibition were detected in the *Gnmt*^*-/-*^ mice, indicating that GNMT activity is the hub of the regulatory network modulated by miR-873-5p.

In the *Mdr2*^*-/-*^ cholestatic mouse model inhibition of miR-873-5p for only 4 weeks starting at the time at which an overt inflammation, cholestasis, and severe fibrosis is observed (4 months old mice), resulted in reduced BA-related cell death and cholangiocyte proliferation, with mild blunted inflammation and decreased fibrosis related markers.

DNA methylation and hydroxymethylation are epigenetic mechanisms that modulate gene expression and play a critical role in the development of liver fibrosis^22,24–26^. We have identified that miR-873-5p changes methyltransferase signaling and DNA methylation state during BDL-mediated hepatic injury and in the chronic model of *Mdr2*^*-/-*^ mice. Anti-miR-873-5p therapy restores the transmethylation flux to normal levels in both animal models of liver damage. This miR-873-5p-dependent modulation of the methylome reduces global DNA methylation by decreasing Dnmt3a expression that functions as a de novo methyltransferase^24^. DNA methylation changes that occur in the livers of mice under anti-miR-873-5p treatment are associated with altered expression of specific genes. Indeed, in BDL mouse, anti-miR-873-5p restores *Pias 1,3* levels, genes highly sensible to methylation changes and regulators of STAT3 phosphorylation with the concomitant regression of the inflammatory response. The reduction in STAT3 activation observed in BDL mouse after anti-miR-873-5p treatment, could also have an important effect in the amelioration of fibrosis, due to its direct role in promoting HSC, the major fibrogenic cell type^37^. In *Mdr2*^*-/-*^ mice, the upregulation of the RASSF tumor suppressor, also susceptible to epigenetic modification linked to miR-873-5p treatment, blocks the Ras/Raf/MEK/ERK pathway, related to tumor formation^38^.

Hepatic fibrosis is also related to methylation changes in BA metabolism and cholangiocyte proliferation^29^. BAs are highly cytotoxic, their synthesis, transport, and pool size are tightly regulated under physiological conditions. *FXR*, implicated in every step of BA homeostasis and susceptible to be regulated by methylation^29^, was upregulated under anti-miR-873-5p treatment. FXR has been shown to induce BSEP and MRP2 in hepatocytes^39^. Importantly, the inhibition of miR-873-5p counteracts the repression of BSEP and the basolateral drug transporters MRP3 and MRP4 in our cholestatic models. Thus, the stimulation of basolateral BA efflux may be an important protective response against the cholestatic liver injury detected in BDL and *Mdr2*^-*/*-^ that could also be regulated by DNA methylation of different genes. Regarding cholangiocyte proliferation, *SOX17* promoter was previously found to be hypermethylated and downregulated in cholangiocarcinoma^31^. In addition, downregulation of *SOX17* is implicated in cholangiocyte damage and fibrosis^40^. MiR-873-5p therapy is able to re-express *Sox17* in both cholestatic animal models potentially regulating cholangiocyte proliferation.

Finally, the activation of HSC to myofibroblast is a key process in liver fibrosis mediated by epigenetic mechanism^41^. We now provide evidence that SAMe flux recovery mediated by anti-miR-873-5p treatment and GNMT rescue reduced EZH2 levels. This histone methyltransferase is one of the major regulators for profibrogenic factors^42^. Moreover, other signatures of histone methylation are observed in the BDL model, including changes in the histone methyltransferase *Ash1*, correlating with methylation of the histone H3K4, which has been related with TGFβ induction of fibrosis^33^.Therefore, it is tempting to hypothesize that changes in global methylation seen after GNMT recovery could exert a negative feedback in the transdifferentiation and activation of HSC.

Our results show that miR-873-5p inhibition directly regulates in vivo BA metabolism, cholangiocyte proliferation and activation and hepatocyte cell death. Modulation of hepatocyte cell death by inhibiting miR-873-5p further reduces inflammatory responses and ameliorates the fibrotic phenotype in diverse liver injury mouse models, possibly associated with epigenetic changes (Fig. 7). Indeed, there is an emerging field in the screening of epi-drugs in the fibrosis disease.

Summing up, the significant inverse correlation between hepatic GNMT and miR-873-5p expression both in cirrhotic and cholestatic patients targeting the miR-873-5p/GNMT axis may provide a novel therapeutic approach to treat liver fibrosis.

## Materials and methods

### Human studies

All the studies were performed in agreement with the Declaration of Helsinki, and with local and national laws. The Human Ethics Committee of Valdecilla Hospital and of the University of Navarra approved the study procedures.

### Cirrhotic patients

#### Cohort 1

Sixteen liver samples from cirrhotic patients (grade F4) of diverse etiologies were included in this study. The characteristics of these patients are summarized in Suppl. Table I. Healthy liver samples (from individuals with normal or minimal changes in the liver) were collected at surgery of digestive tumors or from percutaneous liver biopsy performed because of mild alterations of liver function. All samples were obtained from the Biobank of the University of Navarra (Pamplona, Spain).

#### Cohort 2

Thirty-five serum samples from cirrhotic patients (grade F4) (45.7% women and 54.3% men, mean age 63.9 ± 13.4 years) were included in this study from patients recruited ate the Marqués de Valdecilla University Hospital (MVUH, Santander, Spain). Cirrhosis diagnosis was established by clinical or histological data. Etiology was varied: 43% hepatitis C, 31% alcoholic, 17% autoimmune hepatitis, 6% primary biliary cholangitis and 3% hepatitis B. Serum samples (*n* = 9; five women and four men, mean age 32.4 ± 6.4 years) from healthy volunteer subjects were used as controls and recruited through the Gastroenterology and Hepatology Department of MVUH, Santander, Spain.

### Cholestatic patients

#### Cohort 3

Forty-one serum samples from cholestatic patients (PBC = 36; PSC = 5) with different grade of fibrosis (early = 34; advanced = 7) were included in the study (90% women and 10% men, mean age 59.5 ± 12.2 years). PBC/PSC diagnosis was established in MVUH and based on clinical and biochemical data, immunological markers, imaging, liver histology, and exclusion of other possible causes of liver injury. Healthy human serum samples (*n* = 13) (92% women and 8% men, mean age 45.5 ± 10.5) from subjects with AMA in serum and without abnormal liver test were included and provided by the Gastroenterology and Hepatology Department MVUH, Santander, Spain. Characteristics of these patients are summarized in Suppl. Table II.

#### Cohort 4

Sixty-four liver samples from patients with cholestatic liver disease, PBC (*n* = 60) and PSC (*n* = 4) were included in this study (90% women and 10% men, mean age 51.64 ± 10.65 years). PBC/PSC diagnosis, established in Marqués de Valdecilla University Hospital (MVUH, Santander, Spain), was based on clinical and biochemical data, immunological markers, imaging, liver histology, and exclusion of other possible causes of liver injury. Patients were classified for early or advance fibrosis based on histological analysis and according to Ludwig classification stage (Grade I–IV). Characteristics of these patients are summarized in Suppl. Table III. Healthy human liver samples (*n* = 12; 42% women and 58% men, mean age 63.16 ± 13.01 years) from organ-transplant donor were used as controls and recruited through the Gastroenterology and Hepatology Department of MVUH, Santander, Spain.

### Animal studies

Three-month-old male (C57BL6), *Gnmt w*ild type (WT), *Gnmt*-knockout (*Gnmt*^-/-^) and *Mdr2*^*-/-*^ and *Mdr2*^*WT*^ mice were used^15,43^. Animal procedures were approved by CIC bioGUNE’s Animal Care and Use Committee and the competent authority (Diputación de Bizkaia, Spain).

### In vivo miR-873-5p inhibition after BDL

Three-month-old male *Gnmt* WT and *Gnmt*^-/-^ mice were injected in the tail vein with miRIDIAN microRNA Hairpin Inhibitor anti-miR-873-5p or miR-Control (60 µg/mouse) (Dharmacon, USA) at 3 days after BDL and repeated on day 5 using Invivofectamine 3.0, following the manufacturer’s instructions (Invitrogen, USA). Mice were sacrificed at day 7, blood withdrawn and livers removed and snap frozen in liquid nitrogen or fixed in formalin for subsequent analysis.

### In vivo miR-873-5p inhibition in Mdr2^-/-^ mice

Four-month-old male *Mdr2*^*-/-*^ mice were injected in the tail vein with miRIDIAN anti-miR-873-5p or miR-Control (60 µg/mouse) once a week during 4 weeks using Invivofectamine 3.0, following the manufacturer’s instructions (Invitrogen, USA). Animals were then sacrificed, blood withdrawn and livers were removed and snap frozen in liquid nitrogen or fixed in formalin.

### In vitro miR-873-5p inhibition

Mouse primary hepatocytes were isolated from male *Gnmt* WT mice via collagenase perfusion as described^44^. All adhered cells were maintained in MEM with 10% fetal bovine serum (FBS). NHC cholangiocytes and primary mouse hepatocytes were transfected with anti-miR-873-5p or a miR-Control using DharmaFECT transfection reagent (Dharmacon) following the manufacturer’s procedure.

### SAMe and SAH measurement

Hepatic SAMe and SAH were determined by Liquid-chromatography/mass spectrometry (LC/MS) using a Waters ACQUITY-UPLC system coupled to a Waters Micromass LCT Premier Mass Spectrometer equipped with a Lockspray ionization source as described previously^45^.

### Global DNA methylation measurement

Global DNA methylation (5mC) analyses were performed following the method previously described^46^.

### Bile acids (BA) measurement

Total bile acids in serum of *Mdr2*^*-/-*^ mice were measured using the Total Bile Acid Assay Kit (Cell Biolabs, Inc, USA) following the manufacturer’s instructions.

### Statistical analysis

Data are represented as mean ± SEM. mRNA/RNA data is normalized as fold change vs. control. Statistical significance was determined by the Student’s *t*-test when two groups were compared and when comparing three groups one-way analysis of variance (ANOVA) was used. A *p* < 0.05 was considered significant.

## Electronic supplementary material

Supplemental Material
