# Supplementary material for: MiR-873-5p acts as an epigenetic regulator in early stages of liver fibrosis and cirrhosis
Source: Cell Death Dis. 2018 Sep 20;9(10):958. doi: 10.1038/s41419-018-1014-y (PMC6148053; doi:10.1038/s41419-018-1014-y)
Supplement: Supplementary file 1 — Supplemental Material [file 41419_2018_1014_MOESM1_ESM.doc]

***MiR-873-5p acts as an epigenetic regulator in early stages of liver fibrosis and cirrhosis***

David Fernández-Ramos^1,2,*^, Pablo Fernández-Tussy^1,*^, Fernando Lopitz-Otsoa^1^, Virginia Gutiérrez-de Juan^1^, Nicolás Navasa^1^, Lucía Barbier-Torres^1^, Imanol Zubiete-Franco^1^, Jorge Simón^1^, Agustín F. Fernández^3,4^, Ander Arbelaiz^5^, Ana M. Aransay^1,2^, José Luis Lavín^1^, Naiara Beraza^1^, María J. Perugorria^2,5,6^, Jesus M. Banales^2,5,6^, Erica Villa^7^, Mario F. Fraga^8^, Juan Anguita^1,6^, Matias A Avila^2,9^ Carmen Berasain ^2,9^, Paula Iruzibieta^2,10^, Javier Crespo^2,10^, Shelly C. Lu^11^, Marta Varela-Rey^1,2^, José M. Mato^1,2^, Teresa C. Delgado^1,#^ and María L. Martínez-Chantar^1,2,#^

^1^ CIC bioGUNE, Centro de Investigación Centro de Investigación Cooperativa en Biociencias, Derio, Bizkaia, Spain.

^2^ Centro de Investigación Biomédica en Red de Enfermedades Hepáticas y Digestivas (CIBERehd), Instituto de Salud Carlos III, Madrid, Spain.

^3^ Cancer Epigenetics Laboratory, Institute of Oncology of Asturias (IUOPA), HUCA, University of Oviedo, Oviedo, Asturias, Spain.

^4^ Instituto de Investigación Sanitaria del Principado de Asturias (IISPA), Oviedo, Spain.

^5^ Department of Liver and Gastrointestinal Diseases, Biodonostia Health Research Institute – Donostia University Hospital – University of the Basque Country (UPV/EHU), San Sebastian, Spain.

^6^ Ikerbasque, Basque Foundation for Science, Bilbao, Spain

^7^ Department of Gastroenterology, Azienda Ospedaliero-Universitaria & University of Modena and Reggio Emilia, Modena, Italy.

^8^ Nanomaterials and Nanotechnology Research Center (CINN-CSIC)-Universidad de Oviedo-Principado de Asturias, Spain

^9^ Hepatology Programme, CIMA-University of Navarra, IdiSNA, Pamplona, Spain.

^10^ Department of Gastroenterology and Hepatology, Marqués de Valdecilla University Hospital. Infection, Immunity and Digestive Pathology Group, Research Institute Marqués de Valdecilla (IDIVAL), Santander, Spain.

^11^ Division of Digestive and Liver Diseases, Cedars-Sinai Medical Center, Los Angeles, CA.

**^*^**These authors contributed equally to this work.

**Joint** **Corresponding authors^#^:** Teresa Cardoso Delgado, CIC bioGUNE, Technology Park of Bizkaia, 48160 Derio, Bizkaia, Spain. tcardoso@cicbiogune.es; Tel: +34-944-061318; Fax: +34-944-061301; María Luz Martínez Chantar, CIC bioGUNE, Technology Park of Bizkaia, 48160 Derio, Bizkaia, Spain. mlmartinez@cicbiogune.es; Tel: +34-944-061318; Fax: +34-944-061301.

**Table of contents:**

- Supplemental Material and Methods.
- Supplemental Figures 1-7
- Supplemental Table I-V.

**Supplemental Material And Methods**

**Carbon tetrachloride**

CCl4 (Sigma-Aldrich, St. Louis, MO) was administered by intraperitoneal injection at a dose of 0.6 mL/kg once a week, during 6 weeks.

**Bile Duct Ligation**

Bile-duct ligation (BDL) surgery was performed in animals under isoflurane anesthesia, as described ^1^. Animals were monitored until 72-hours after surgery and given non-steroidal analgesic drugs each 24-hours.

**Ammonia measurement**

The ammonia in the liver of *Mdr2^-/-^*mice was measured by a morphological method for ammonia detection in the liver as published before ^2^.

**MicroRNA quantitative real-time PCR**

RT-PCR was performed for miR-873-5p following a TaqMan® MicroRNA Reverse Transcription Kit (Life Technologies, USA) procedure using 50 ng of total RNA. qPCR was performed with the TaqMan Universal PCR Master Mix No AmpErase UNG kit following manufacturer’s procedure. miR-873-5p expression levels were normalized to the U6 snRNA.

**Serum microRNA quantification.**

MicroRNAs were isolated from serum with the miRNeasy Serum/Plasma Kit (Qiagen, Germany) following manufacturer’s procedure, adding miR-39 as Spike-In Control. RT/q-PCR were performed as described. miR-873-5p levels were normalized to the Spike-In.

**Luciferase reporter assay**

*Gnmt* cDNA sequence was purchased from Source biosciences (cDNA clone MGC:13738 IMAGE:4210236). The 3’UTR sequence was sub-cloned into the pmirGLO vector (Promega, USA) to obtain the pmirGLO-*Gnmt*-3’UTR plasmid.

Primary hepatocytes were transfected with the pmirGLO or pmirGLO-*Gnmt*-3’UTR vectors together with miRIDIAN microRNA Hairpin Mimic miR-873-5p or scrambled siRNA using the DharmaFECT Duo Transfection Reagent (Dharmacon). Firefly/Renilla luciferase activities were determined in cell lysates with the Dual-GLO luciferase assay system (Promega). Normalized data were calculated as luciferase/Renilla ratio. Detailed vector sequence is indicated:

**IRAV23H07 *Mus musculus* glycine N-methyltransferase, mRNA (cDNA clone MGC:13738 IMAGE:4210236), complete cds**

t ccatgtgctc aagaagacag actgagtttc tccggctccc agaagcccat gctcaggcaa tggcccctac cctaagacca tcccctaatg cagatattgc atttgggtgc agatgtgggg gtcgggcaaa cggagtaaac aatacagtct gca

**Protein Isolation & Western Blotting**

Protein Isolation & Western Blotting Extraction of total protein from cultured cells and livers was performed as described ^3^. 2-30 µg of protein were electrophoresed on sodium dodecyl sulfatepolyacrylamide gels and transferred onto membranes. Further information provided upon request.

**RNA Isolation and Quantitative Real-Time PCR**

RNA was isolated with Trizol (Invitrogen), and its concentration and integrity were determined. PCRs were performed using iQ™ SYBR® Green Supermix (Biorad) and the Bio-Rad iCycler thermocycler (BioRad, Hercules, CA). The Ct values were extrapolated to a standard curve, and then normalized to the housekeeping expression (*ARP* and *GAPDH*). Further information provided upon request.

**RNAseq**

Starting from 700ng of total RNA, mRNAseq libraries were obtained with TruSeq RNA Sample Preparation v2 kit (Illumina Inc., Cat.# RS-122-2001) and sequenced in a HiScanSQ (Illumina Inc.). Resulting single-reads of 50nt were mapped against the mouse (*mm10*) reference genome by *Tophat* ^4^ program to account for spliced junctions. Outliers, when present, were filtered after performing Hierarchical Clustering and Principal Component Analysis to check the correlation between samples from each group. Resulting *BAM* alignment files were the input for the Differential Expression (DE) analysis, carried out by *DESeq2* ^5^, to detect differentially expressed genes among sample groups. GO enrichment was tested using the *goseq* ^6^ and GOdb ^7^ bioconductor package and *PantherDB* ^8^ database. **ACCESSION NUMBER**: the NCBI Gene Expression Omnibus accession number for the RNA-seq data reported in this paper is **GSE77840**.

**Apoptosis Measurement**

Caspase 3 activity assay was performed as previously described ^3^.

**TUNEL assay**

TUNEL assay was performed in primary hepatocytes using the *in situ* cell death detection kit (Roche) according to the manufacturer’s instructions.

**Immunohistochemistry**

Paraffin embedded liver samples were sectioned, dewaxed and hydrated. Immunohistochemistry was performed as previously described ^9^.

**Supplemental Figures:**


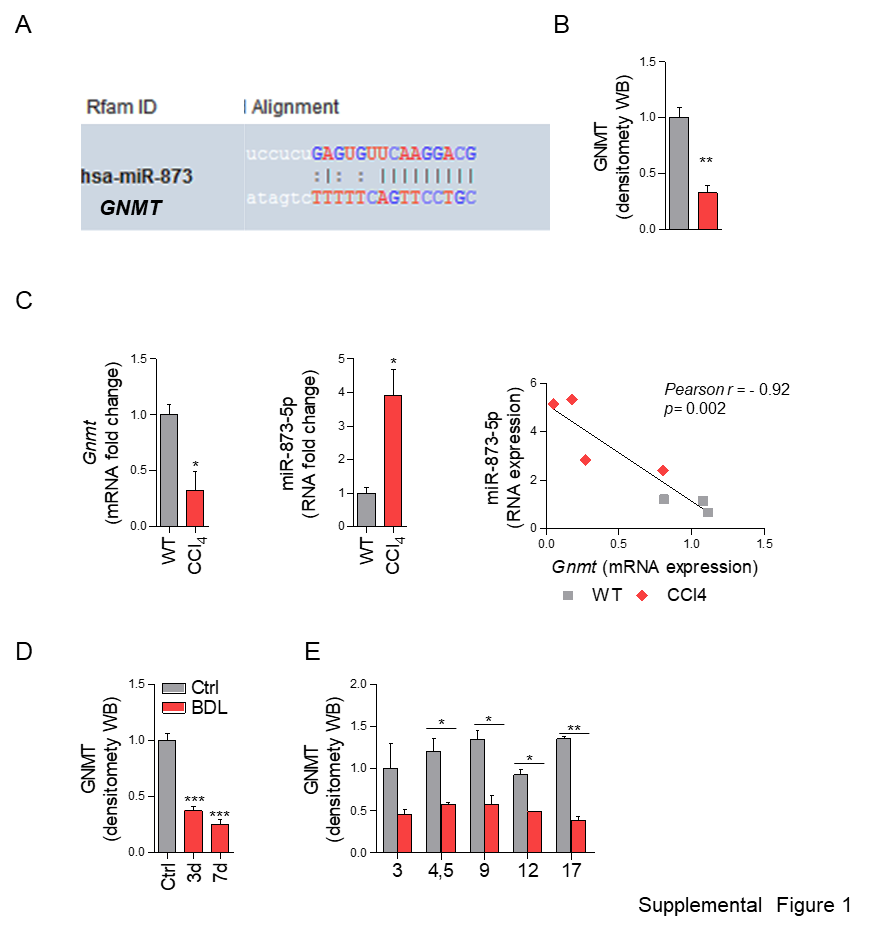


**Supplemental Figure 1**

(A) Expected targeting of *GNMT* mRNA by microRNA-873 obtained from TargetScan database. (B) Densitometry analysis of GNMT after mimic-miR-873-5p transfection. (N=3). (C) *GNMT*-miR-873-5p expression and correlation in control mice and after six weeks of CCl_4_ administration (N=4). (D) Densitometry analysis of GNMT in control and BDL mice at different time points. (E) Densitometry analysis of GNMT in *Mdr2^WT^* and *Mdr2^-/-^* mice at indicated age. Data normalized as fold change vs. control. Error bars represent the means ± SEM. Statistical significance was determined by the Student’s t test or ANOVA when more than 2 groups were compared. p<0.05 *; p<0.01 **; p<0.001 ***.

**
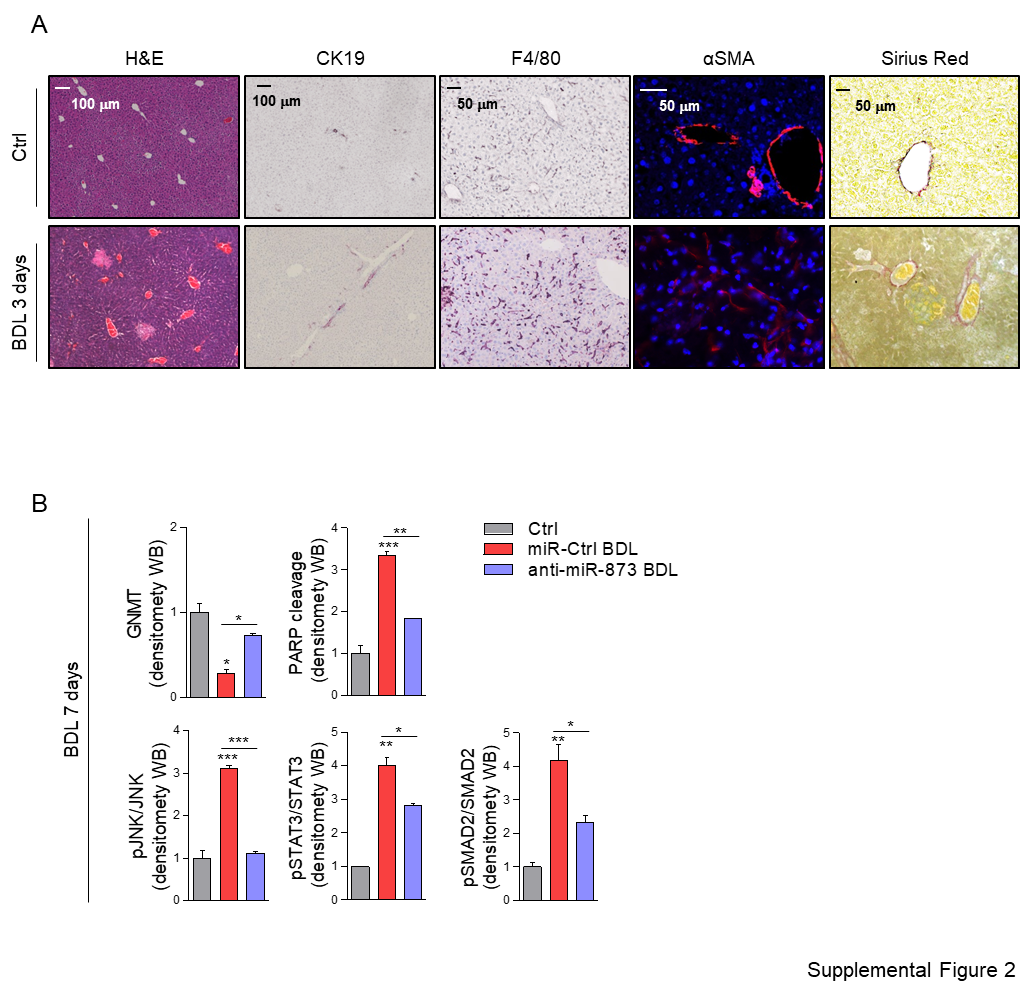
**

**Supplemental Figure 2. Anti-miR-873-5p reduces proinflammatory and profibrogenic gene expression in BDL.**

(A) Liver characterization of the Ctrl and BDL mouse at 3 days post-surgery by IHC analysis. (B) Densitometry analyses of WB of indicated protein vs. indicated loading control in total liver of BDL-Ctrl and anti-miR-873 BDL mice at 7 days (N=4). Data normalized as fold change vs. control. Error bars represent the means ± SEM. Statistical significance was determined by the Student’s t test or ANOVA when more than 2 groups were compared. p<0.05 *; p<0.01 **; p<0.001 ***.

**
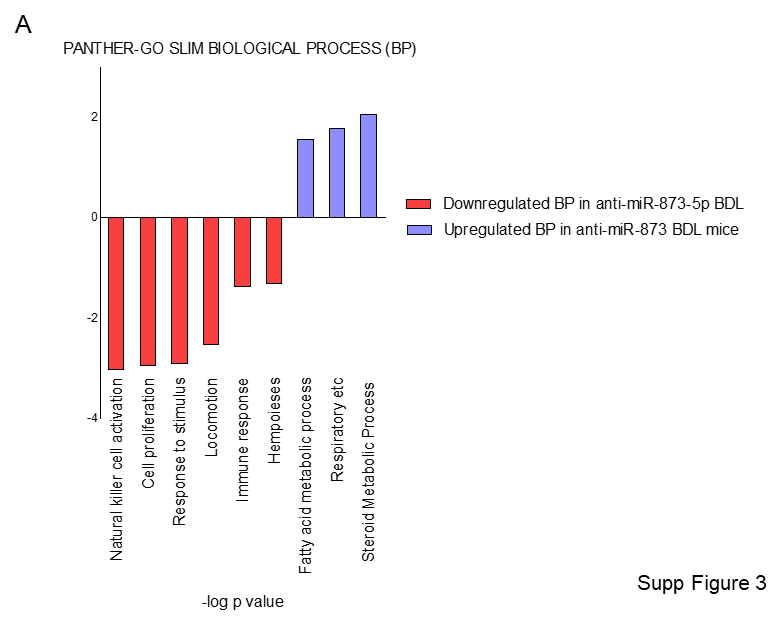
**

**Supplemental Figure 3. MiR-873-5p is implicated in the BDL-induced inflammatory and immune response.**

(A) Fold enrichment analysis of significantly overrepresented biological processes in miR-Ctrl vs. anti-miR-873-5p mice (red) and in anti-miR-873-5p vs. miR-Ctrl (blue) at 7 days of BDL (N=4).


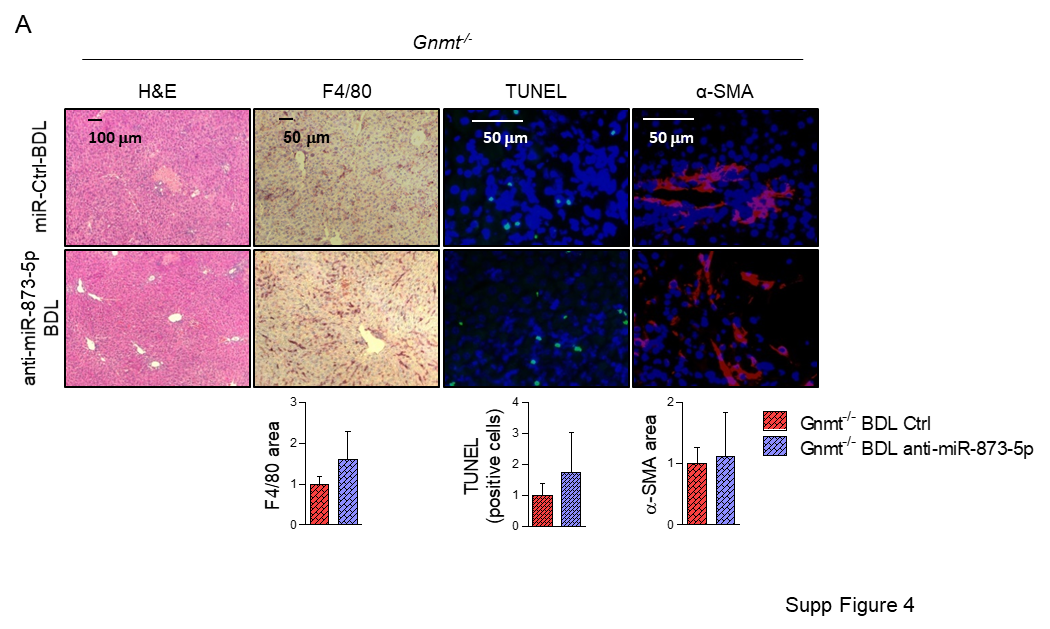


**Supplemental Figure 4. miR-873-5p inhibition in *Gnmt*^-/-^ mice indicates the specificity of miR-873 targeting *Gnmt* in BDL.**

(A) H&E, F4/80, Tunel and αSMA staining in liver sections from miR-Ctrl and miR-873-5p-inhibited-mice at 7 days of BDL in *Gnmt*^-/-^ mouse. Data normalized as fold change vs. control (N=4). Error bars represent the means ± SEM.


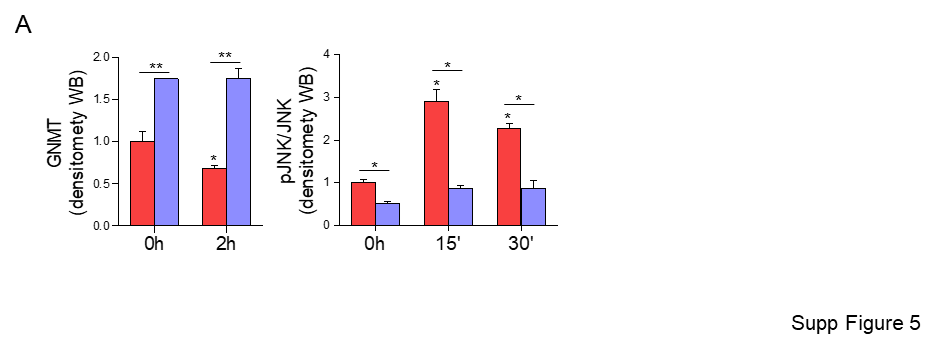


**Supplemental Figure 5. MiR-873-5p inhibition reduces bile acid induced apoptosis recovering GNMT levels in the hepatocytes.**

(A) Densitometry analyses of WB of indicated protein vs. indicated loading control in primary hepatocytes after 2h of DCA and anti-miR.873-5p treatment (N=3). Data normalized as fold change vs. control. Error bars represent the means ± SEM. Statistical significance was determined by the Student’s t test p<0.05 *; p<0.01 **.


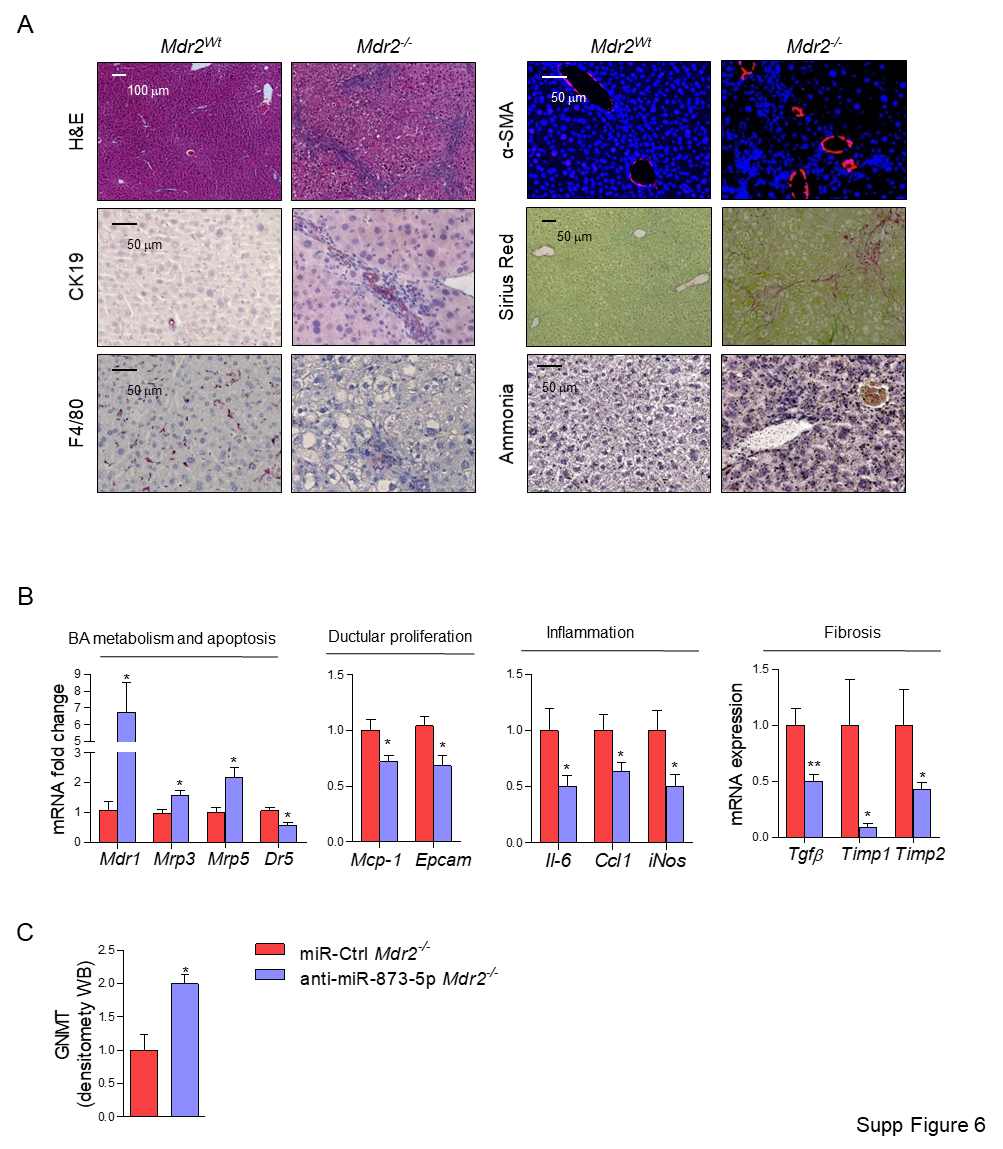


**Supplemental Figure 6. MiR-873-5p inhibition ameliorates bile acid-induced liver damage in the *Mdr2*^-/-^ mouse.**

(A) Liver characterization of four months old *Mdr2^-/-^*mouse by IHC analysis. (B) qPCR analysis of indicated genes implicated in bile acid metabolism, inflammation and profibrogenic processes in total liver extracts of *Mdr2^-/-^* miR-Ctrl and anti-miR-873-5p mice. (C) Densitometry analyses of WB of indicated protein vs. indicated loading control. Data normalized as fold change vs. control. Error bars represent the means ± SEM (N=5). Statistical significance was determined by the Student’s t test p<0.05 *; p<0.01 **; p<0.001 ***.


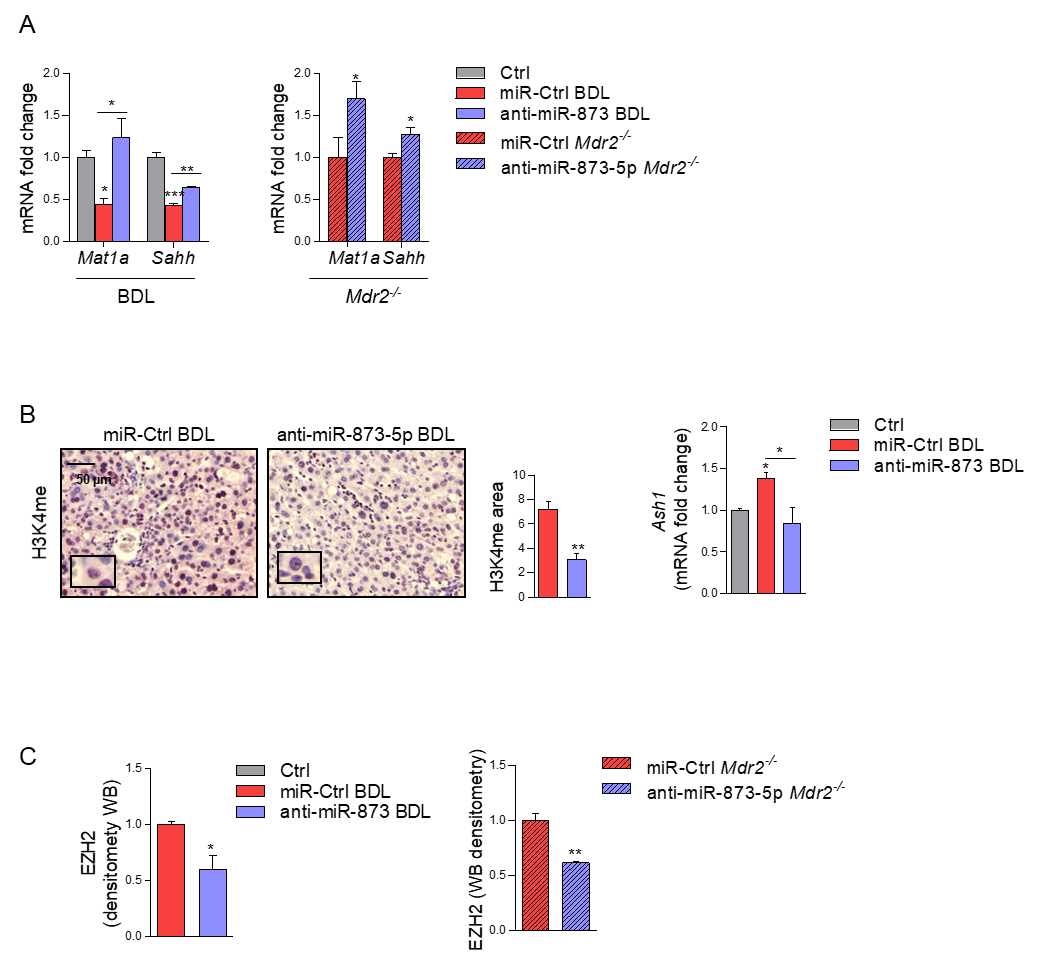


**Supplemental Figure 7. MiR-873-5p inhibition affects SAMe metabolism enzymes.**

(A) qPCR analysis of indicated genes implicated in SAMe metabolism in total liver extracts. (B) H3K4 staining in liver sections and qPCR analysis of the histone methyltransferase *Ash1* in miR-Ctrl and miR-873-5p-inhibited-mice at 7 days of BDL. (C) Densitometry analyses of WB of indicated protein vs. indicated loading control. Data normalized as fold change vs. control. Error bars represent the means ± SEM. Statistical significance was determined by the Student’s t test or ANOVA when more than 2 groups were compared. p<0.05 *; p<0.01 **; p<0.001 ***.

**Supplemental Table I. Characterization of liver cirrhotic patients**

| **VARIABLE** |  | **CIRRHOSIS** | **HEALTHY** |
| --- | --- | --- | --- |
| **N** |  | 16 | 10 |
| **Age (years, mean, min-max)** |  | 68 (60-75) | 61.7 54-78 |
| **Gender (F/M)** |  | 2 / 16 | 2 / 8 |
| **Etiology** |  |  |  |
|  | HBV  HCV | 3 (19%)  5 (31%) |  |
|  | Alcohol | 5 (31%) |  |
|  | Others | 3 (19%) |  |
| **Child Pugh Class** |  |  |  |
|  | A | 6 (37.5%) |  |
|  | B | 6 (37.5%) |  |
|  | C | 4 (25%) |  |
| **HCC** |  |  |  |
|  | YES | 7 (44%) |  |
|  | NO | 9 (56%) |  |

**Supplemental Table II. Characteristics of serum cholestatic patients.** Primary biliary cholangitis (PBC); primary sclerosing colangitis (PSC); alanine aminotransferase (AST); alkaline phosphatase (ALP); bilirubin (BI).

| **Variable** | **Early stage** | **Advanced stage/cirrhosis** | **Control subjects** |
| --- | --- | --- | --- |
| **n** | 34 | 7 | 13 |
| **Age (years)** | 60.5 ± 11.5 | 55. ± 15.3 | 45.5 ± 10.5 |
| **Gender (f/m)** | 33 / 1 | 4 / 3 * | 12 / 1 |
| **PBC/PSC** | 33 / 1 | 3 / 4 * | - |
| **AST (u/l)** | 33.3 ± 25 | 56.1 ± 27.7* | 18.4 ± 3.2 |
| **ALP (u/l)** | 131.4 ± 65. 8 | 256.2 ± 178.8 | 71 ± 21.5 |
| **Bi (mg/dl)** | 0.6 ± 0.3 | 1.5 ± 1.1 | 0.33 ± 0.1 |
| **Albumin (g/dl)** | 4.2 ± 0.2 | 3.9 ± 0.4 * | 4.4 ± 0.2 |
| **Fibroscan (transient elastography kPa)** | 5.8 ± 2.2 | 20.2 ± 15.2 * | 3.8 ± 0.6 |

**Supplemental Table III. Characterization of liver cholestatic patients**

| **VARIABLE** | **CHOLESTATIC PATIENTS** |
| --- | --- |
| N | 64 |
| Age (years, mean±SD) | 51.64 ± 10.65 |
| Gender (F/M) | 57 / 7 |
| Early stages, n (%) | 47 (73.44) |
| Grade I | 20 |
| Grade I-II | 12 |
| Grade II | 15 |
| Advanced stages, n (%) | 17 (26.56) |
| Grade II-III | 3 |
| Grade III | 3 |
| Grade IV | 11 |
| Cirrhosis, n (%) | 11 (17.19) |

**Supplemental Table IV. Downregulated genes found within the most important overrepresented processes in anti-miR-873-5p-BDL vs, miR-Ctrl-BDL mice**

| **LOCOMOTION** | **CELL PROLIFERATION** | **NATURAL KILLER CELLS** | **IMMUNE RESPONSE** |
| --- | --- | --- | --- |
| SERUM AMYLOID A-1 PROTEIN  (PTHR23424:SF5) | Protein Gm13277  Gm13290 ortholog | Protein Gm13277  Gm13290 ortholog | Protein Gm13277  Gm13290 ortholog |
| C-X-C motif chemokine 9  Cxcl9 ortholog | Protein Gm13277  Gm13290 ortholog | T-cell-interacting, activating receptor on myeloid cells protein 1  Tarm1 ortholog | Protein Gm13277  Gm13290 ortholog |
| Serum amyloid A-2 protein  Saa2 ortholog | Protein Gm13277  Gm13290 ortholog | Protein Gm13277  Gm13290 ortholog | T-cell-interacting, activating receptor on myeloid cells protein 1  Tarm1 ortholog |
| C-X-C motif chemokine 10  Cxcl10 ortholog | C-X-C motif chemokine 9  Cxcl9 ortholog | Secreted frizzled-related protein 2  Sfrp2 ortholog | Protein Gm13277  Gm13290 ortholog |
|  | Protein Gm13276  Gm13276 ortholog | Protein Gm13276  Gm13276 ortholog | Secreted frizzled-related protein 2  Sfrp2 ortholog |
|  | Protein Ifnz  Ifnz ortholog | Protein Ifnz  Ifnz ortholog | Protein Gm13276  Gm13276 ortholog |
|  | C-X-C motif chemokine 10  Cxcl10 ortholog | Protein Gm13277  Gm13290 ortholog | Protein Ifnz  Ifnz ortholog |
|  | Protein Gm13277  Gm13290 ortholog | Oxidized low-density lipoprotein receptor 1  Olr1ortholog | Protein Gm13277  Gm13290 ortholog |
|  | Protein Gm13277  Gm13290 ortholog | Protein Gm13277  Gm13290 ortholog | Guanylate-binding protein 5  Gbp5 ortholog |
|  | Protein Gm13277  Gm13290 ortholog | Protein Gm13277  Gm13290 ortholog | Oxidized low-density lipoprotein receptor 1  Olr1 ortholog |
|  |  |  | C-C motif chemokine 7  Ccl7 ortholog |
|  |  |  | Protein Gm13277  Gm13290 ortholog |
|  |  |  | Protein Gm13277  Gm13290 ortholog |

**Supplemental Table V. Upregulated genes found within the most important overrepresented processes in anti-miR-873-5p-BDL vs. miR-Ctrl-BDL** mice.

| **STEROID METABOLIC PROCESS** | **RESPIRATORY ELECTRON TRANSPORT CHAIN** | **FATTY ACID METABOLIC PROCESS** | **GENERATION OF PRECURSOR METABOLITES AND ENERGY** |
| --- | --- | --- | --- |
| MCG141487 Cyp4a32 ortholog | MCG141487 Cyp4a32 ortholog | MCG141487 Cyp4a32 ortholog | MCG141487 Cyp4a32 ortholog |
| Cholesterol 7-alpha-monooxygenase Cyp7a1 ortholog | Cholesterol 7-alpha-monooxygenase Cyp7a1 ortholog | Acyl-CoA synthetase short-chain family member 3, mitochondrial Acss3 ortholog | Cholesterol 7-alpha-monooxygenase Cyp7a1 ortholog |
| Cytochrome P450 4A14 Cyp4a14 ortholog | Cytochrome P450 4A14 Cyp4a14 ortholog | Cytochrome P450 4A14 Cyp4a14 ortholog | Cytochrome P450 4A14 Cyp4a14 ortholog |
| Cytochrome P450 4A10 Cyp4a10 ortholog | Cytochrome P450 4A10 Cyp4a10 ortholog | Cytochrome P450 4A10 Cyp4a10 ortholog | Cytochrome P450 4A10 Cyp4a10 ortholog |
| Protein Cyp4a31 Cyp4a31 ortholog | Protein Cyp4a31 Cyp4a31 ortholog | Protein Cyp4a31 Cyp4a31 ortholog | Protein Cyp4a31 Cyp4a31 ortholog |

**References**

1. Zubiete-Franco, I. *et al.* Deregulated neddylation in liver fibrosis. *Hepatol. Baltim. Md* **65,** 694–709 (2017).

2. Gutiérrez-de-Juan, V. *et al.* A morphological method for ammonia detection in liver. *PloS One* **12,** e0173914 (2017).

3. Embade, N. *et al.* Murine double minute 2 regulates Hu antigen R stability in human liver and colon cancer through NEDDylation. *Hepatol. Baltim. Md* **55,** 1237–1248 (2012).

4. Trapnell, C., Pachter, L. & Salzberg, S. L. TopHat: discovering splice junctions with RNA-Seq. *Bioinforma. Oxf. Engl.* **25,** 1105–1111 (2009).

5. Love, M. I., Huber, W. & Anders, S. Moderated estimation of fold change and dispersion for RNA-seq data with DESeq2. *Genome Biol.* **15,** 550 (2014).

6. Young, M. D., Wakefield, M. J., Smyth, G. K. & Oshlack, A. Gene ontology analysis for RNA-seq: accounting for selection bias. *Genome Biol.* **11,** R14 (2010).

7. GO.db. *Bioconductor* Available at: http://bioconductor.org/packages/GO.db/. (Accessed: 28th April 2017)

8. Thomas, P. D. *et al.* PANTHER: a library of protein families and subfamilies indexed by function. *Genome Res.* **13,** 2129–2141 (2003).

9. Barbier-Torres, L. *et al.* Stabilization of LKB1 and Akt by neddylation regulates energy metabolism in liver cancer. *Oncotarget* **6,** 2509–2523 (2015).
